# Supplementary material for: Monitoring phagocytic uptake of amyloid β into glial cell lysosomes in real time
Source: Chem Sci. 2021 Jul 21;12(32):10901–18. doi: 10.1039/d1sc03486c (PMC8372545; doi:10.1039/d1sc03486c)
Supplement: SC-012-D1SC03486C-s001 [file SC-012-D1SC03486C-s001.pdf]

Electronic Supplementary Material (ESI) for Chemical Science.
This journal is © The Royal Society of Chemistry 2021

SUPPORTING INFORMATION

Monitoring phagocytic uptake of amyloid β into glial cell lysosomes in real time

Priya Prakash,[a†] Krupal P Jethava,[a†] Nils Korte,[b†] Pablo Izquierdo,[b†] Emilia Favuzzi,[cd] Indigo VL Rose,[e] Kevin A Guttenplan,[f] Palak Manchanda,[a] Sayan Dutta,[g] Jean-Christophe Rochet,[gh] Gord Fishell,[cd] Shane A Liddelow,[eji] David Attwell,[b] Gaurav Chopra[ahklm*]

[†]These authors contributed equally to this work.

[a]Department of Chemistry, Purdue University, West Lafayette, IN 47907, USA.
[b]Department of Neuroscience, Physiology and Pharmacology, University College London, London WC1E 6BT, UK.
[c]Department of Neurobiology, Harvard Medical School, 220 Longwood Avenue, Boston, MA 02115, USA.
[d]Stanley Center at the Broad, 75 Ames Street, Cambridge, MA 02142, USA.
[e]Neuroscience Institute, NYU Grossman School of Medicine, New York, NY 10016, USA.
[f]Department of Neurobiology, Stanford University, Stanford, CA 94305, USA.
[g]Department of Medicinal Chemistry and Molecular Pharmacology, Purdue University, West Lafayette, IN 47907, USA.
[h]Purdue Institute for Integrative Neuroscience, Purdue University, West Lafayette, IN 47907, USA.
[i]Department of Neuroscience & Physiology, NYU Grossman School of Medicine, New York, NY 10016, USA.
[j]Department of Ophthalmology, NYU Grossman School of Medicine, New York, NY 10016, USA.
[k]Purdue Institute for Drug Discovery, 720 Clinic Drive, West Lafayette, IN 47907, USA.
[l]Purdue Center for Cancer Research, Purdue University, West Lafayette, IN 47907, USA.
[m]Purdue Institute of Inflammation, Immunology and Infectious Disease, Purdue University, West Lafayette, IN 47907, USA.

[*]Corresponding author:
Gaurav Chopra, PhD
720 Clinic Dr., West Lafayette, IN 47907
+1 (765) 496-6108
gchopra@purdue.edu

INSTRUMENTATION USED FOR CHEMICAL CHARACTERIZATION OF $A\beta^{pH}$

1. MALDI-MS spectra were obtained using Applied Biosystems Voyager DE PRO instrument (Main parameters: Number of laser shots: 100/spectrum, Laser intensity: variable 2500 to 3300, Laser repetition rate: 20.0 Hz, accelerating voltage 25000 V).
2. ^{1}H-NMR spectra were obtained in DMSO-d_6 solvent using a Bruker AV-III-500-HD 500 MHz NMR instrument.
3. ATR-FTIR spectra were recorded using Thermo Fisher Nicolet FTIR instrument.
4. AFM images were recorded using Veeco Multimode instrument with NanoScope V controller

SUPPORTING MOVIES

Movie S1 https://youtu.be/2gmwNXQ1nsc **- Phagocytosis of $A\beta^{pH}$ by HMC3 cells**. Phagocytic uptake of $A\beta^{pH}$ by HMC3 cells. The green fluorescent intensity within the cells increases over time with increased $A\beta^{pH}$ uptake. The cell at the center of the frame engulfs a neighboring cell (during the 5 hour time point) by forming a phagocytic cup (seen at the 4 hour time point).

Movie S2 https://youtu.be/jQW0Aj1B8sI **- Phagocytosis of $A\beta^{pH}$ by BV2 cells.** Phagocytic uptake of $A\beta^{pH}$ by BV2 microglia is confirmed by the appearance of green fluorescence within the acidic phagosomes of the cells. A change from the ramified state to the amoeboid state is observed visually during this process.

Movie S3 https://youtu.be/kjOWvxxd3do **- Phagocytosis of $A\beta^{pH}$ by N9 cells.** Phagocytic uptake of $A\beta^{pH}$ by N9 microglia demonstrated by the appearance of green fluorescence within the cells.

Movie S4 https://youtu.be/yn1NCeJZrqI **- *In vivo* imaging of $A\beta^{pH}$ in mouse cortex.** *In vivo* two-photon imaging of the barrel cortex before and after topical application of $A\beta^{pH}$ over an imaging period of 22.5 minutes (intensity enhanced).

Movie S5 https://youtu.be/AiY_09Mdc3Q **- *In vivo* imaging of $A\beta^{pH}$ in mouse cortex.** *In vivo* two-photon imaging of the barrel cortex before and after topical application of $A\beta^{pH}$ over an imaging period of 43.5 minutes. The fluorescence increase in cell somata indicates $A\beta^{pH}$ uptake.

SUPPLEMENTAL TEXT, FIGURES AND FIGURE LEGENDS

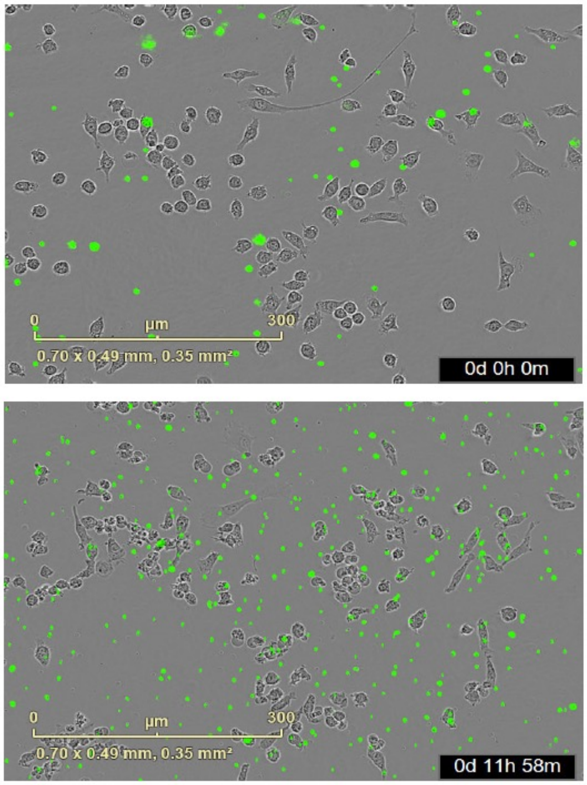

Figure S1A. Live cell imaging of 1 µm fluorescent latex beads (Sigma-Aldrich, #L1030) applied to BV2 cells for 24 hours (~ 0, 6, 12, and 24 hours). It is difficult to determine if the beads are within or outside the cell bodies.

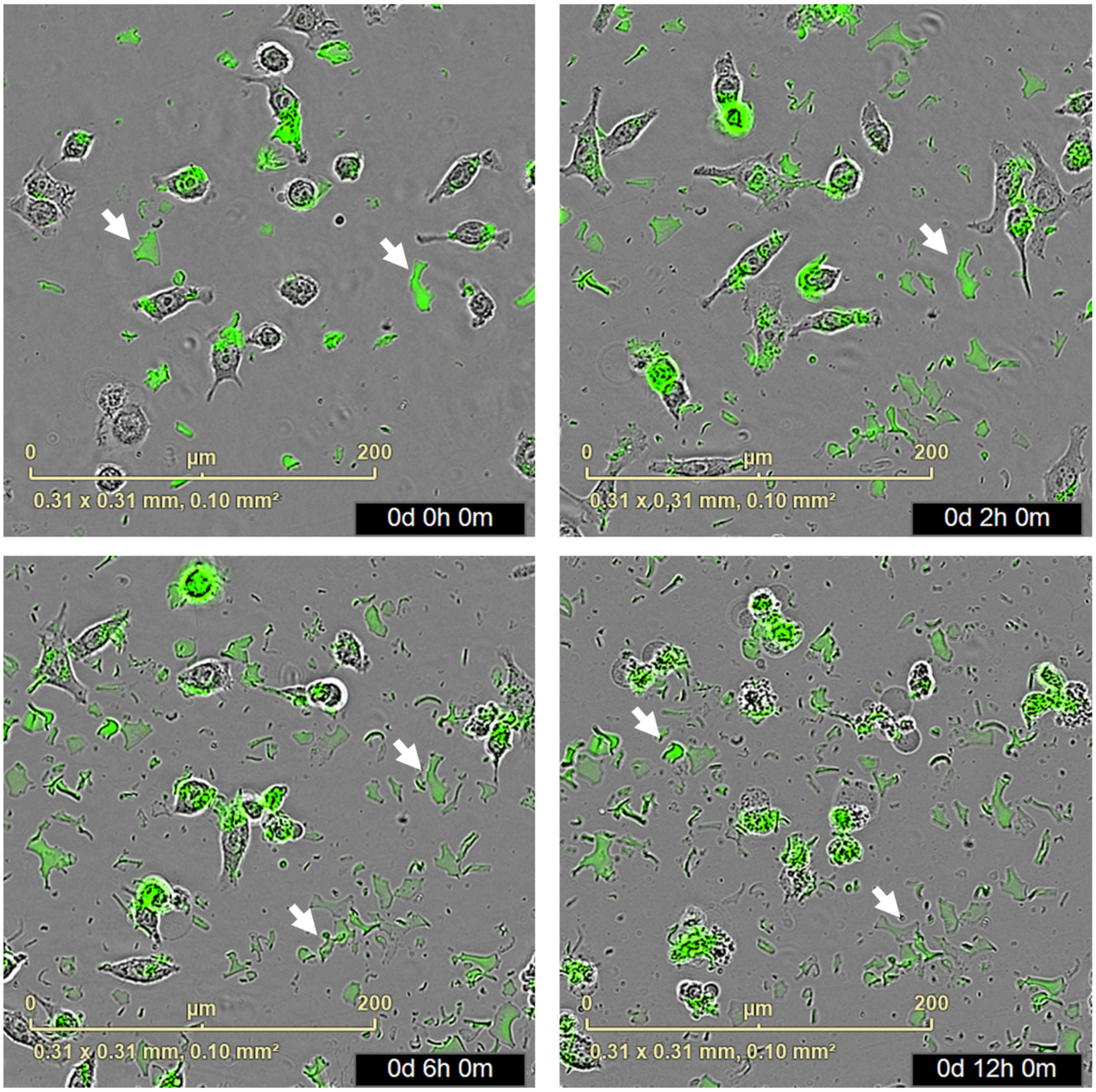

Figure S1B. Live cell imaging of BV2 mouse microglia treated with fluorescein-labeled Aβ peptides (AnaSpec, Inc. #23525-05) at 0, 2, 6, and 12 hour time points. High background noise is observed in addition to non-specific fluorescence in and around the microglial cells.

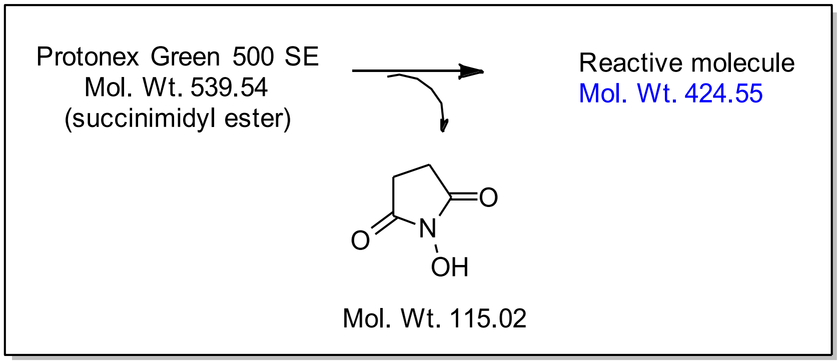

Figure S2A. The mass calculation for the reactive part of Protonex Green 500, SE after conjugation with an amine functional group of Aβ1-42. The Protonex Green 500, SE is a succinimidyl ester of active dye molecule (molecular weight 539.54) and the leaving group *N*-hydroxy succinimide has a molecular weight of 115.02. Therefore, the reactive fragment has a molecular weight of 424.55.

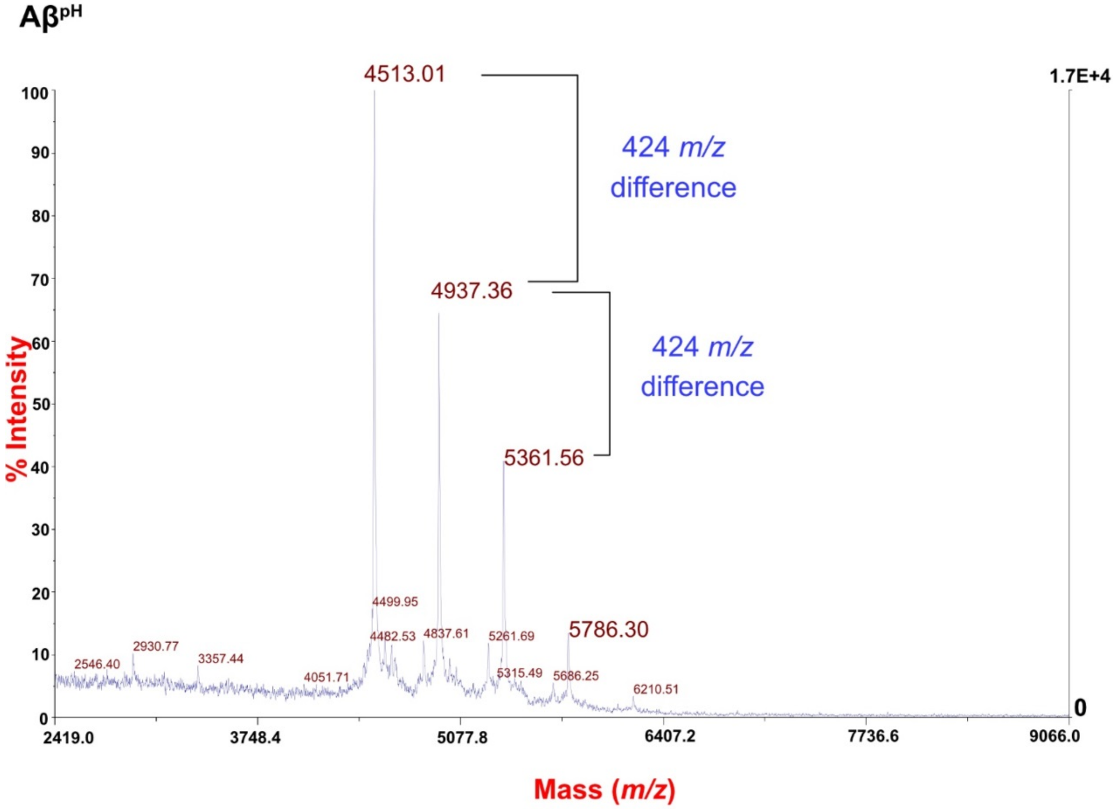

Figure S2B. MALDI-MS spectrum [%intensity *vs.* Mass (*m/z*)] for Aβ-Protonex Green conjugate (Aβ^{pH}) corresponding to *m/z* of 5357.06 and 4932.87. The *m/z* difference of 424 indicates the addition of reactive fragment of Protonex Green 500, SE upon conjugation with an amine functional groups present in Aβ1-42.

Proton nuclear magnetic resonance (^1H-NMR) spectrum shows a chemical shift for protons. A chemical shift is a relative resonant frequency to a standard magnetic field. Chemical shift (δ) is usually expressed in parts per million (ppm). The position and number of chemical shifts are used to determine the structure and functional groups present in a molecule. For example, chemical shifts for aliphatic protons are in 0.5 to 5.0 ppm region and for aromatic protons are in 6.5 to 9 ppm region depending upon the electrochemical environment of protons. This basis was used to identify aliphatic and aromatic protons in the ^1H-NMR spectrum of PTXG, $A\beta_{1-42}$, PTXG-$A\beta^{pH}$ shown in **Figures S3A-S3C**, respectively.

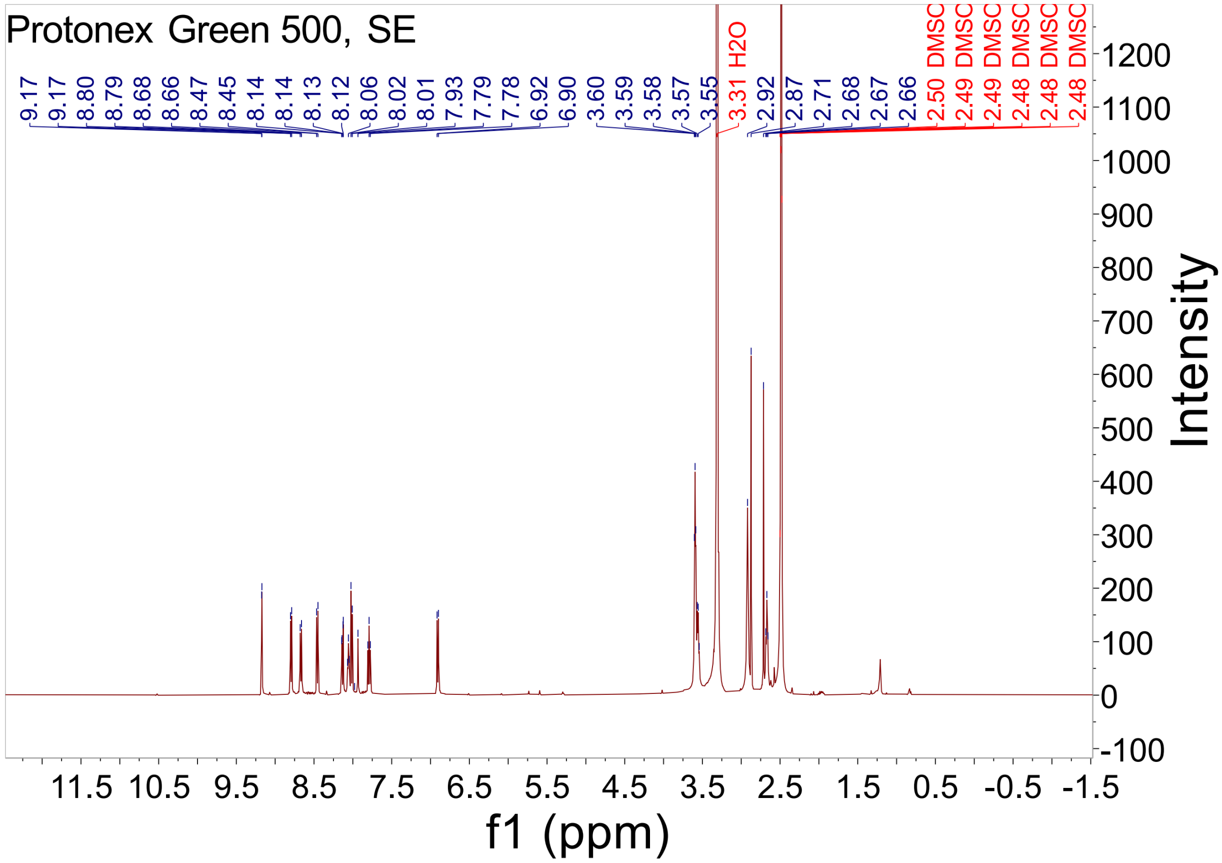

Figure S3A. ^1H-NMR spectrum [Intensity *vs.* ppm] of the Protonex Green 500, SE in DMSO-d_6. shows the presence of aliphatic (2.0 to 4.0 ppm region) and aromatic protons (6.5 to 9.5 ppm region to chemically characterize the material used for the reaction.

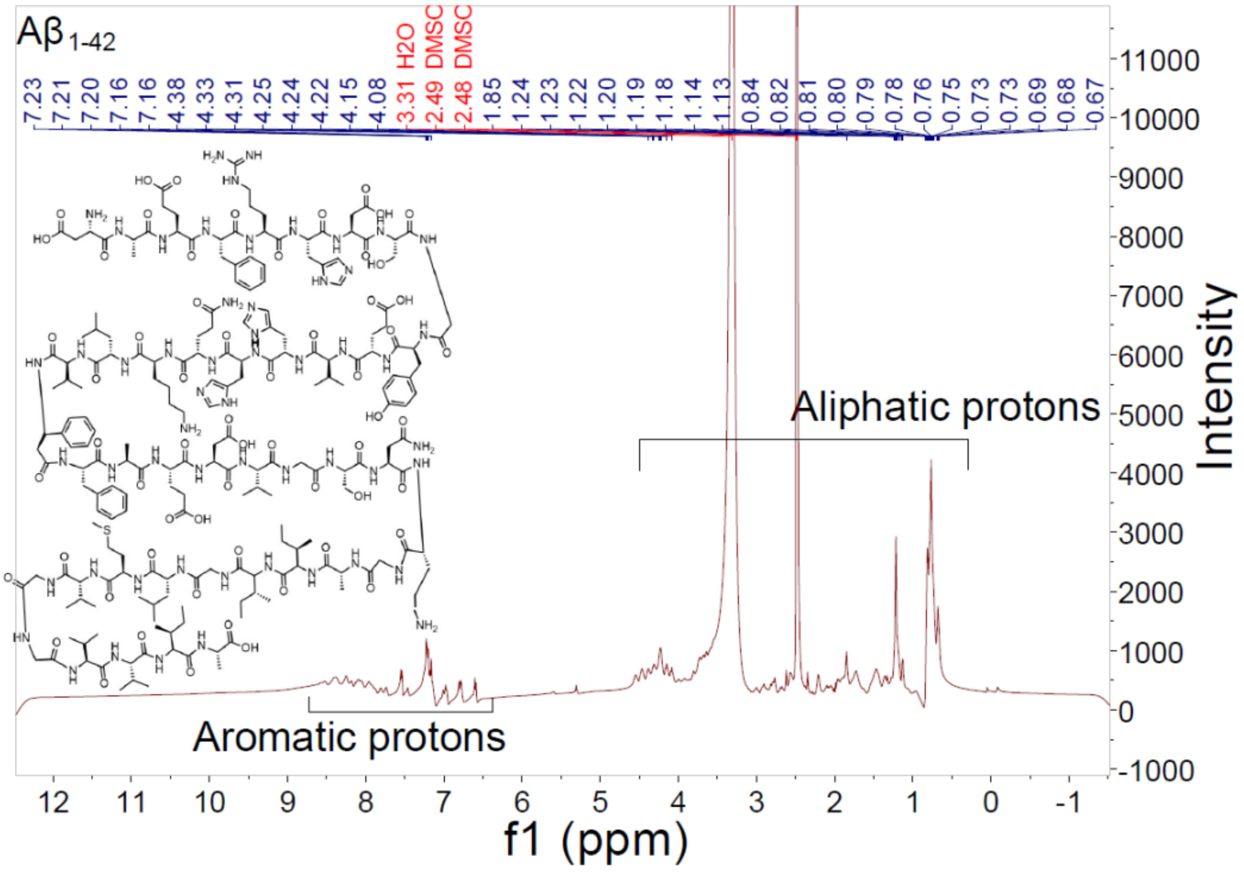

Figure S3B. ^{1}H-NMR spectrum [Intensity *vs.* ppm] of A$\beta_{1\text{-}42}$ in DMSO-d_6. The A$\beta_{1\text{-}42}$ peptide has amino acids containing aromatic (phenyl, 4-hydroxyphenyl, imidazolyl rings) and aliphatic (side chains and peptide backbone) functional groups. The ^{1}H-NMR spectrum of A$\beta_{1\text{-}42}$ confirms the presence of protons arising from aliphatic and aromatic functional group-containing amino acids to characterize the material used for the reaction.

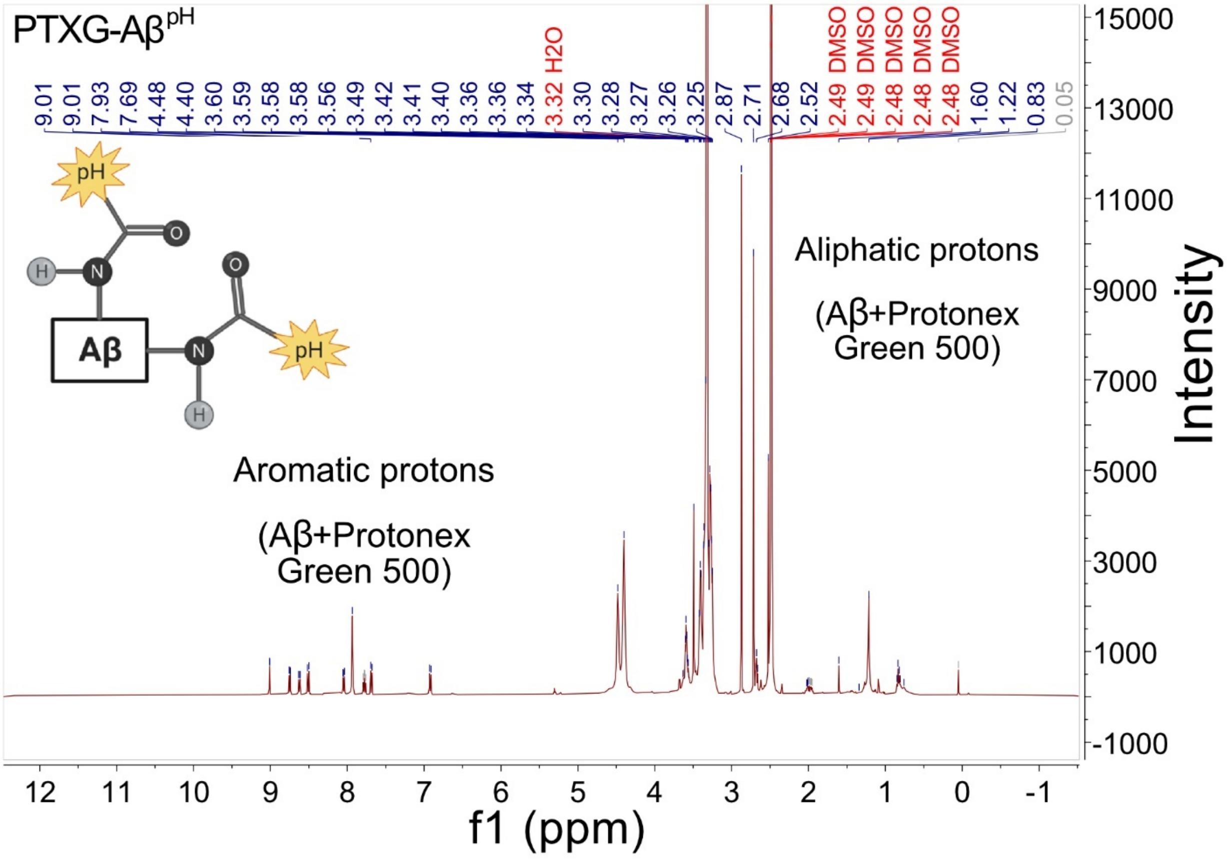

Figure S3C. ^{1}H-NMR spectrum [Intensity *vs.* ppm] of the synthetic Protonex Green-conjugated Aβ (PTXG-Aβ^{pH}) in DMSO-d_6. The spectrum exhibits peaks at aliphatic and aromatic regions that are present due to functional groups present in both Protonex Green 500 and A$\beta_{1\text{-}42}$ (compared to **Figures S3A, S3B**) confirms the conjugation of Protonex Green 500 with A$\beta_{1\text{-}42}$.

Fourier Transform InfraRed (FTIR) spectroscopy utilizes the frequencies associated with the bonds in a molecule that typically vibrate around 4000 cm^{-1} to 400 cm^{-1}, known as the Infrared region of the electromagnetic spectrum. This region is associated with specific frequencies that change the vibration patterns of chemical bonds, resulting in an FTIR spectrum. A typical FTIR spectrum is visualized in a graph of infrared light absorbance (or transmittance) on the y-axis *vs.* frequency or wavenumber (cm^{-1}) on the x-axis. In general, the FTIR-spectrum is unique for an individual chemical entity and a change in structure or a functional group can be identified by comparing changes in these spectra. For example, carbonyl (C=O) is a functional group that is easily identified by FTIR spectroscopy due to a prominent bond stretching vibration peak at unique wavenumber range of around 1670-1820 cm^{-1}. Since carbonyl (C=O) groups are present on carboxylic acids, aldehydes, amides, anhydrides, esters, and ketones, unique wavenumbers in FTIR spectrum confirm these specific types of carbonyl groups. Furthermore, chemical modification results in a change in wavenumber to identify changes between specific carbonyl group types. This basis was used to identify chemical changes in the Attenuated Total Reflection Fourier Transform InfraRed (ATR-FTIR) spectrum of $A\beta_{1-42}$, PTXG, PTXG-$A\beta^{pH}$ shown in **Figures S4A-S4B**, respectively.

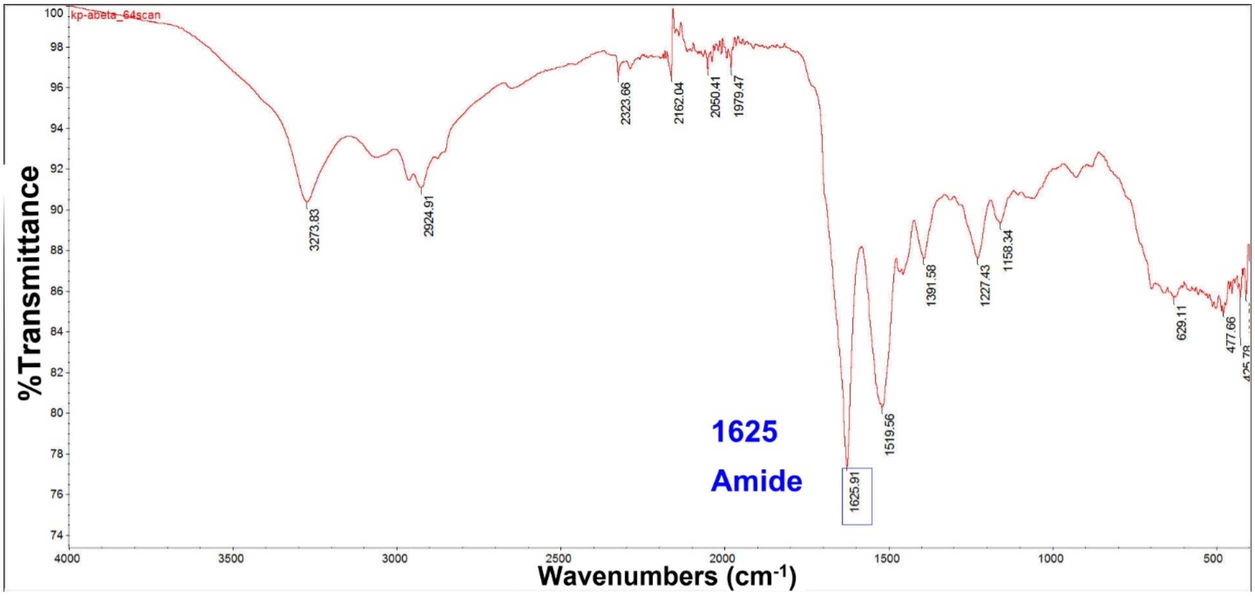

Figure S4A. ATR-FTIR spectrum [%Transmittance *vs.* Wavenumbers (cm^{-1})] of the $A\beta_{1-42}$. The ATR-FTIR shows the intense signal at 1625 cm^{-1} corresponding to carbonyl (C=O) stretching frequencies in amide bonds of the peptide.

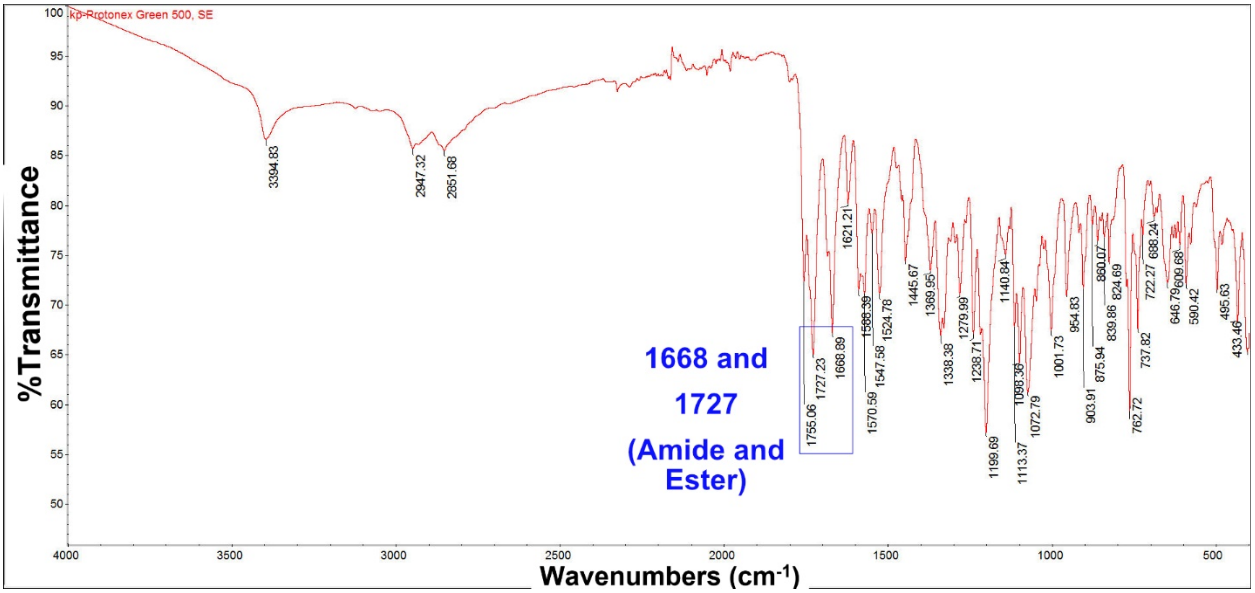

Figure S4B. ATR-FTIR spectrum [%Transmittance *vs.* Wavenumbers (cm^{-1})] of the Protonex Green 500, SE (PTXG). The PTXG chemical structure has amide and ester functional groups and the spectrum shows expected two intense signals at 1668 and 1727 cm^{-1} corresponding to carbonyl (C=O) stretching frequencies in amide and ester functional groups, respectively.

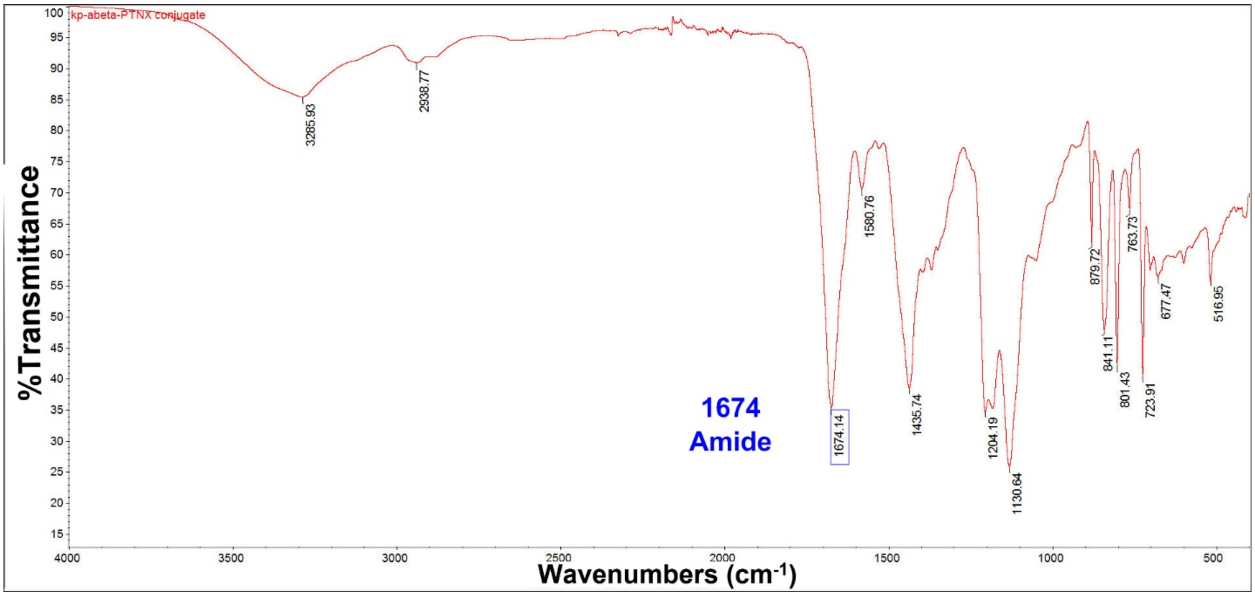

Figure S4C. ATR-FTIR spectrum [%Transmittance *vs.* Wavenumbers (cm^{-1})] of the PTXG-Aβ conjugate (PTXG-$A\beta^{pH}$) shows the change in stretching frequencies of carbonyl (C=O) of the amide functional group compared to **Figures S4A-S4B**. No peak at 1727 cm^{-1} of the ester functional group as shown in **Figure S4B**, suggests a change in the chemical structure of PTXG because of amide bond formation with an amine functional group of $A\beta_{1-42}$.

Taken together **Figures S4A-S4C** show chemical characterization of unconjugated Aβ, PTXG and conjugated PTXG-Aβ using ^1H-NMR and ATR-FTIR spectroscopy.

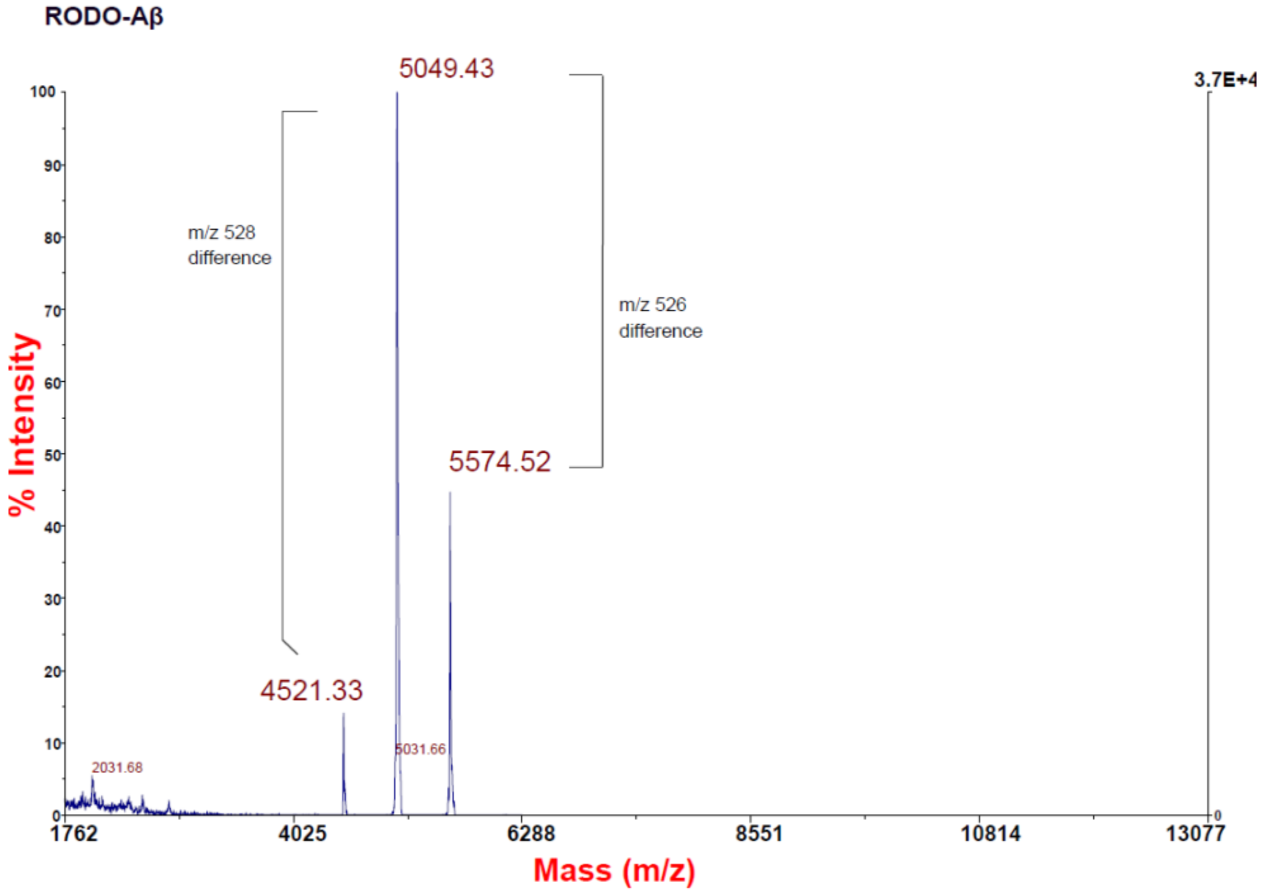

Figure S5. MALDI-MS spectrum [%intensity *vs.* Mass (*m/z*)] for pHrodo Red conjugate of Aβ (RODO-$A\beta^{pH}$) corresponding to *m/z* of 5049.43 and 5574.42. These *m/z* peaks are emerging from the conjugation of pHrodo Red (molecular weight of reactive fragment ~527) with an amine functional group of $A\beta_{1-42}$.

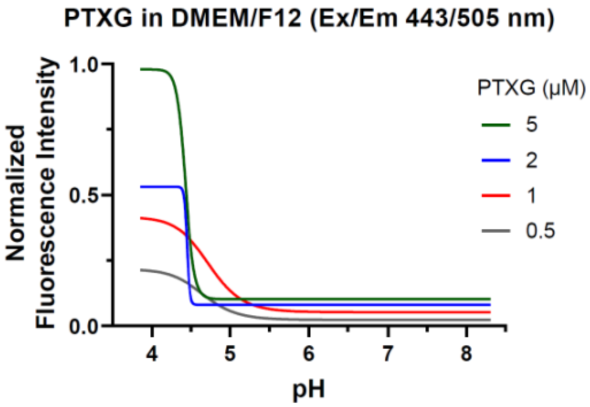

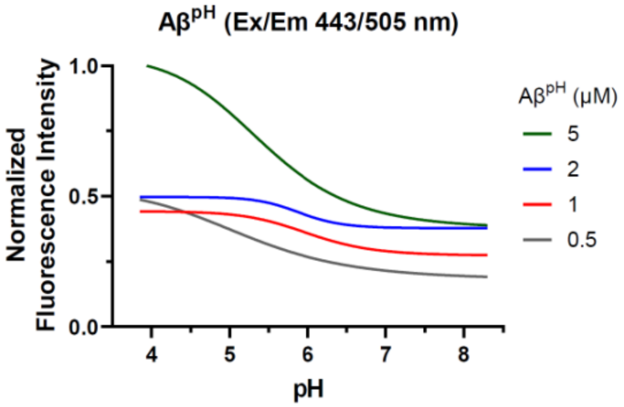

**PTXG Conc. in DMEM/F12 media**	5 μM	2 μM	1 μM	0.5 μM
**pKa**	4.4	4.4	4.7	4.6

**PTXG-Aβ (Aβ^{pH}) Conc.**	5 μM	2 μM	1 μM	0.5 μM
**pKa**	5.4	5.8	5.9	5.2

Figure S6A. pKa of Protonex Green 500, SE® (PTXG) and Aβ^{pH}. Emission spectrum of PTXG and Aβ^{pH} at concentration 5.0, 2.0, 1.0, 0.5 μM in a 384-well plate with 40 μL/well total volume in different pH solutions (pH range 3.85, 4.03, 4.26, 4.46, 4.88, 5.02, 5.30, 5.67, 6.22, 6.44, 6.94, 7.40, 8.31) prepared in DMEM/F12 cell culture medium. Ex/Em is 443/505 nm. The fluorescence intensity was normalized by diving each value by the highest value obtained for 5 μM concentration. pKa was calculated using GraphPad Prism software. pKa value at each concentration is shown in the table. Legend shows colors for concentrations of PTXG and Aβ^{pH} (μM).

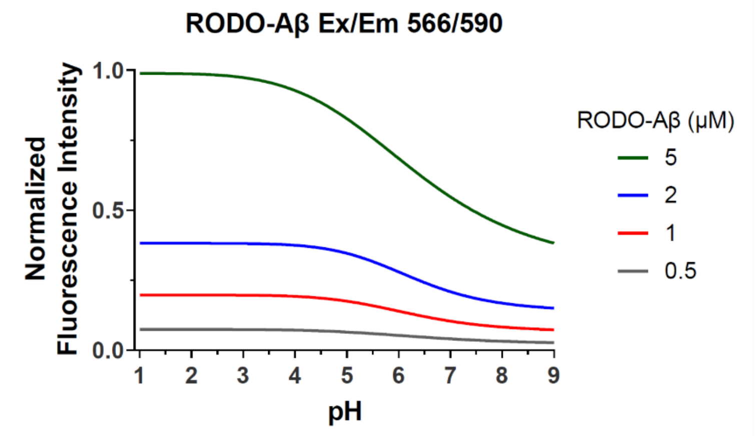

**RODO-Aβ Conc.**	5 µM	2 µM	1 µM	0.5 µM
**pKa**	6.3	6.3	6.2	6.4

Figure S6B. pKa of RODO-Aβ. Emission spectrum of PTXG and $A\beta^{pH}$ at concentration 5.0, 2.0, 1.0, 0.5 µM in a 384-well plate with 40 µL/well total volume in different pH solutions (pH range 3.85, 4.03, 4.26, 4.46, 4.88, 5.02, 5.30, 5.67, 6.22, 6.44, 6.94, 7.40, 8.31) prepared in DMEM/F12 cell culture medium. Ex/Em is 566/590 nm. The fluorescence intensity was normalized by diving each value by the highest value obtained for 5 µM concentration. pKa was calculated using GraphPad Prism software. pKa value at each concentration is shown in the table. Legend shows colors for concentrations of RODO-Aβ (µM).

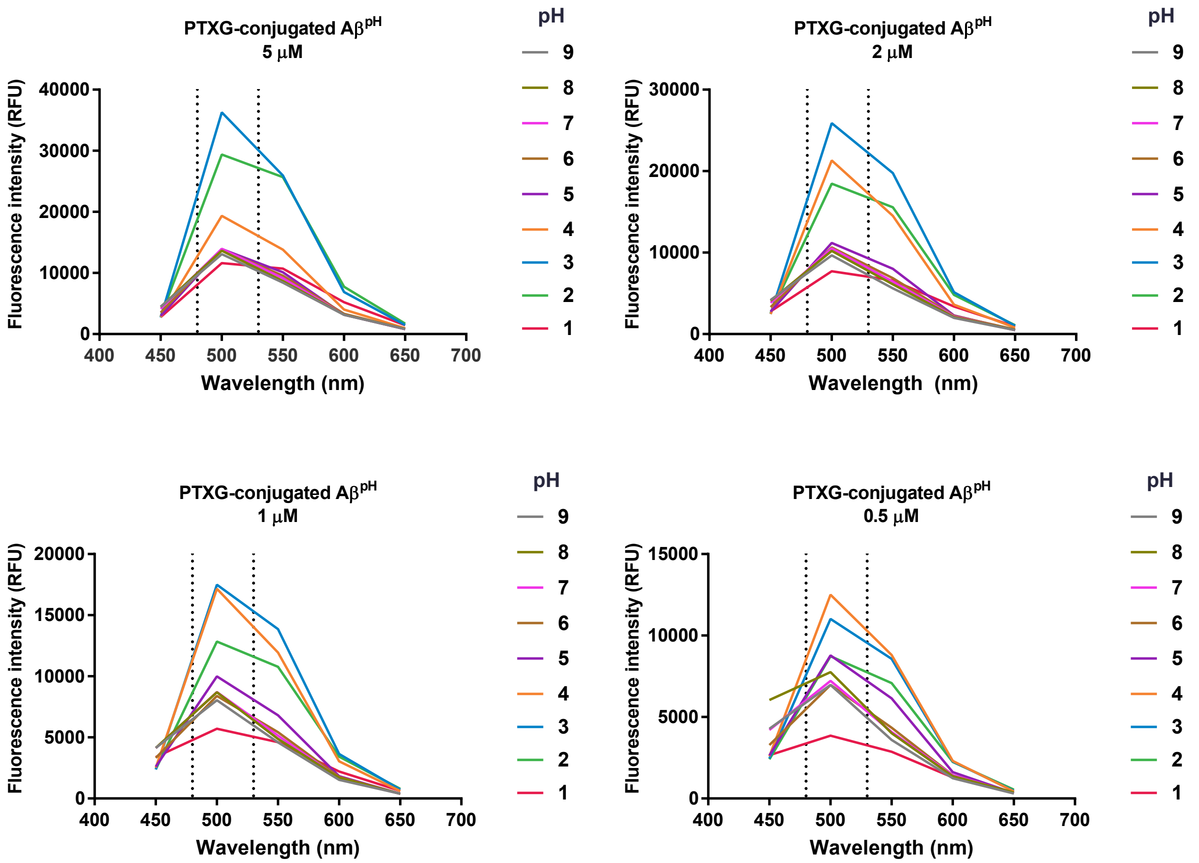

Figure S7A. pH-dependent emission spectra of Protonex Green (PTXG) conjugated Aβ at various concentrations. The emission spectra of the PTXG-$A\beta^{pH}$ reporter showing larger fluorescence intensity for acidic pH and limited fluorescence at the physiological pH of 7.4. Dotted lines highlight the region of maximum emission. Legend shows color code for pH 1 to 9.

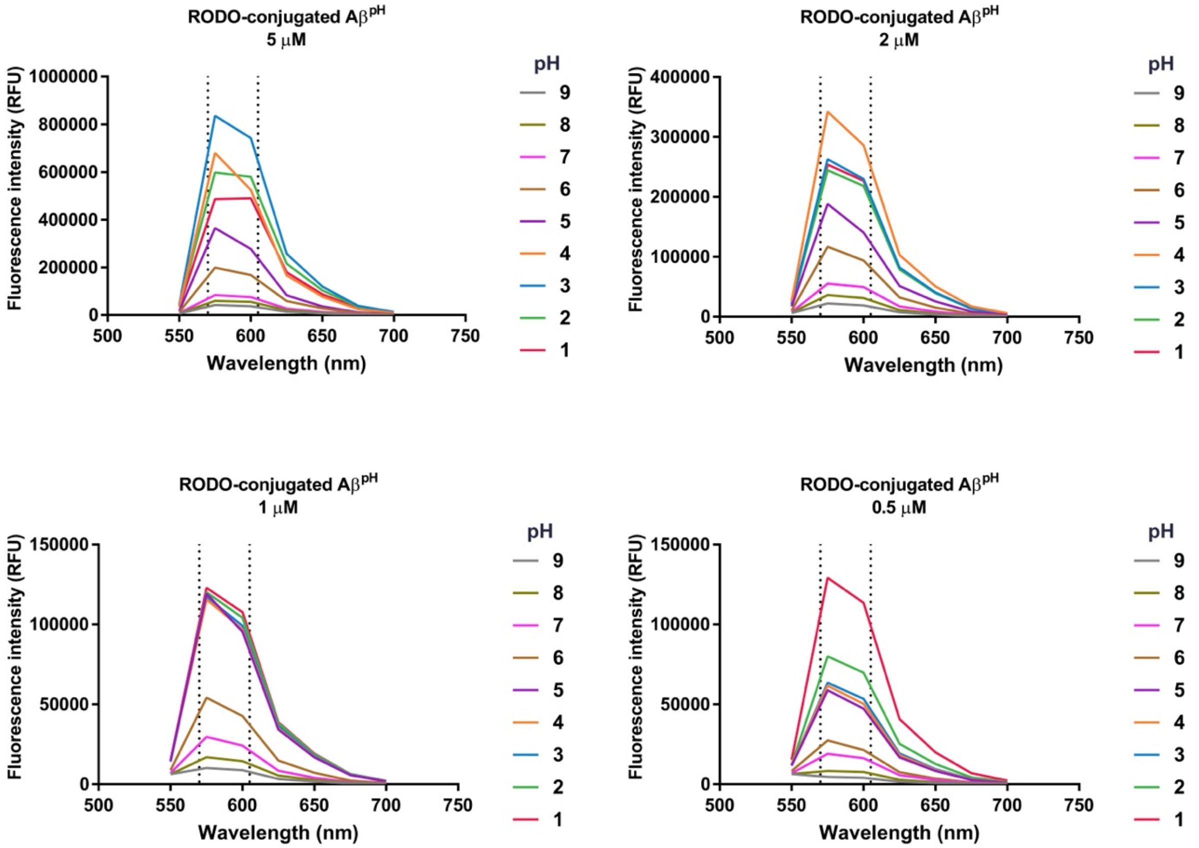

Figure S7B. pH-dependent emission spectra of pHrodo Red (RODO) conjugated Aβ at various concentrations. The emission spectra of the RODO-$A\beta^{pH}$ reporter showing maximum fluorescence over a large pH range of 1.0 to 5.0 along with differences between concentrations. Dotted lines highlight the region of maximum emission. Legend shows color code for pH 1 to 9.

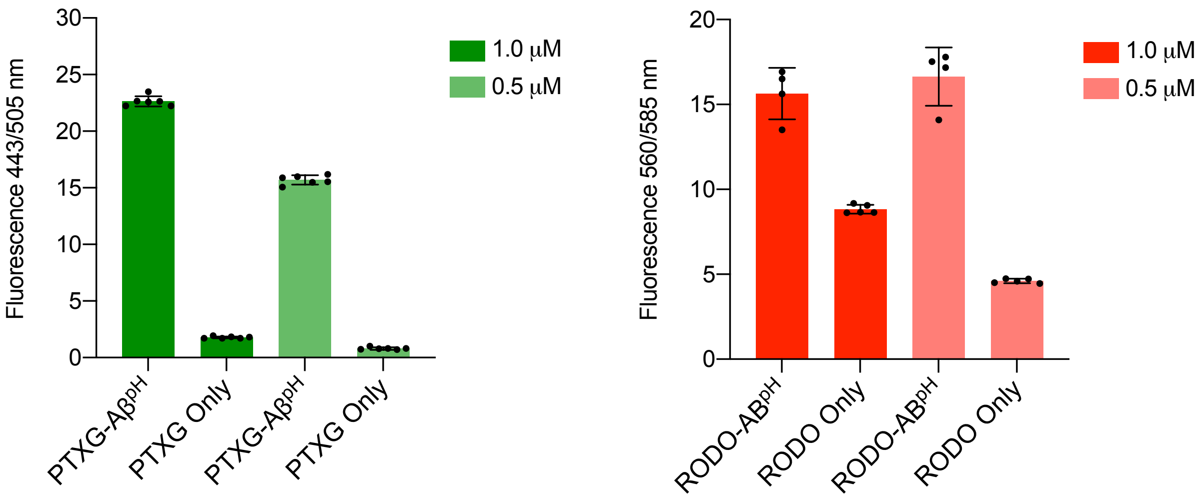

Figure S8. Background fluorescence of PTXG and RODO in cells. BV2 microglia were treated with 1.0 μM or 0.5 μM of PTXG, PTXG-Aβ^{pH}, RODO, and RODO-Aβ^{pH} for 2 hours and fluorescence measured using a microplate reader. Microglial cells treated with PTXG alone showed very low florescence indicating less uptake of the dye alone compared to cells treated with PTXG-Aβ^{pH}. Cells treated with RODO alone showed high background uptake (almost 50% florescence compared to the cell treated with RODO-Aβ^{pH} at higher concentrations) indicating high background of this dye (media fluorescence subtracted from all).

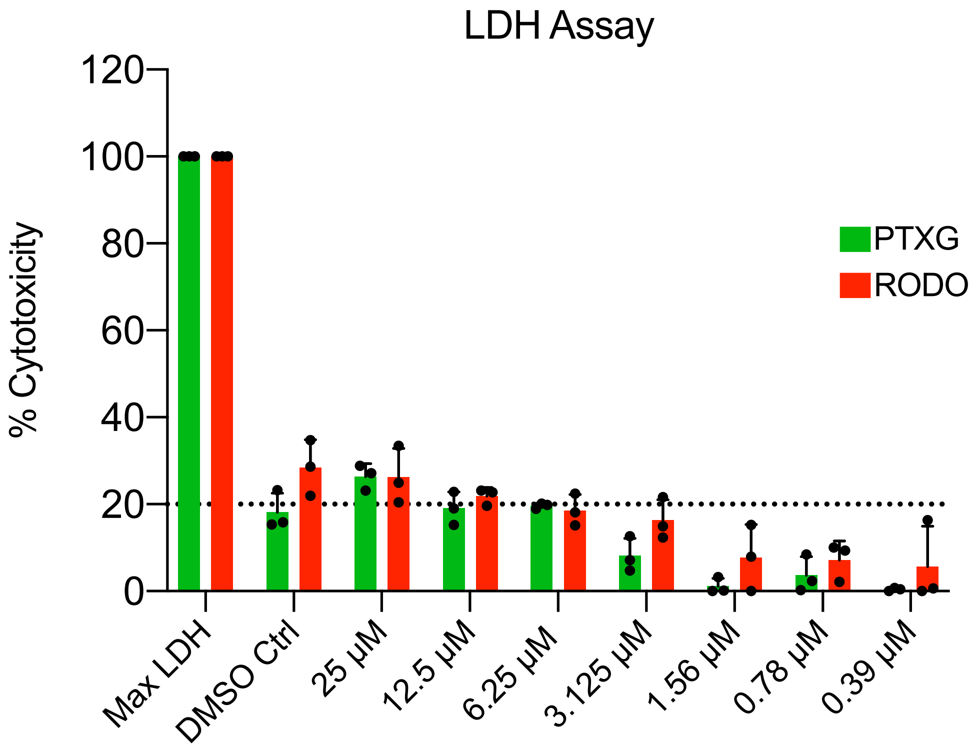

Figure S9. Toxicity of PTXG and RODO dyes. BV2 microglia were treated with different concentrations of PTXG and RODO for 24 hours. Lactate dehydrogenase (LDH) assay was performed per manufacturer's instructions (CyQUANT LDH Cytotoxicity Assay; Thermo #C20300) to determine the toxicity of PTXG and RODO on microglia. Both PTXG and RODO dyes exhibited very low to almost no toxicity to BV2 microglial cells (24 hrs treatment). At the highest dose tested (25 µM), both dyes showed around 30% cytotoxicity. However, at 12.5 µM and below, the dyes showed 20% (dotted line) or less cytotoxicity.

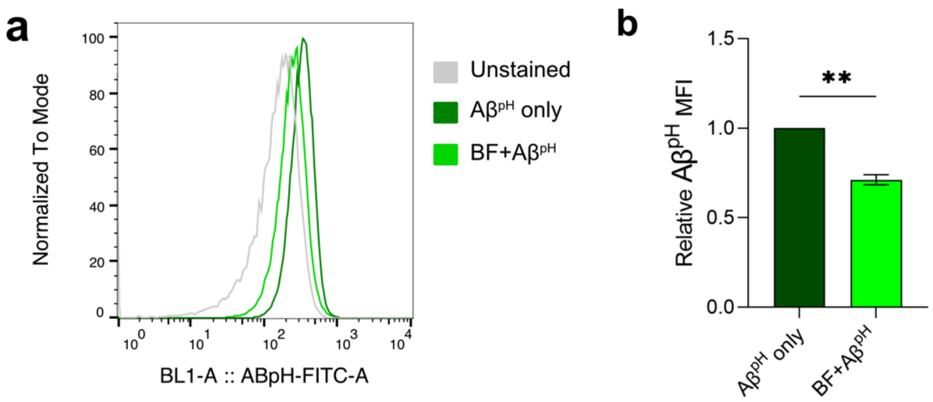

Figure S10. Effect of Bafilomycin-A1 (BF) on fluorescence of Aβ^{pH} in the cells. BV2 microglia were treated with or without 400 nM BF for 1 hour and the fluorescence intensity of Aβ^{pH} measured via flow cytometry. Microglial cells showed a decrease in Aβ^{pH} fluorescence under the BF treatment compared to cells without the influence of BF. (a) A shift in the Aβ^{pH} fluorescence peak towards the left under BF treatment (light green) is seen compared to the fluorescence peak of Aβ^{pH}-only samples (dark green). (b) The corresponding quantification of the median fluorescence intensities (MFI) of the two treatment groups. N=2 biological replicates; unpaired t-test, **p=0.0047.

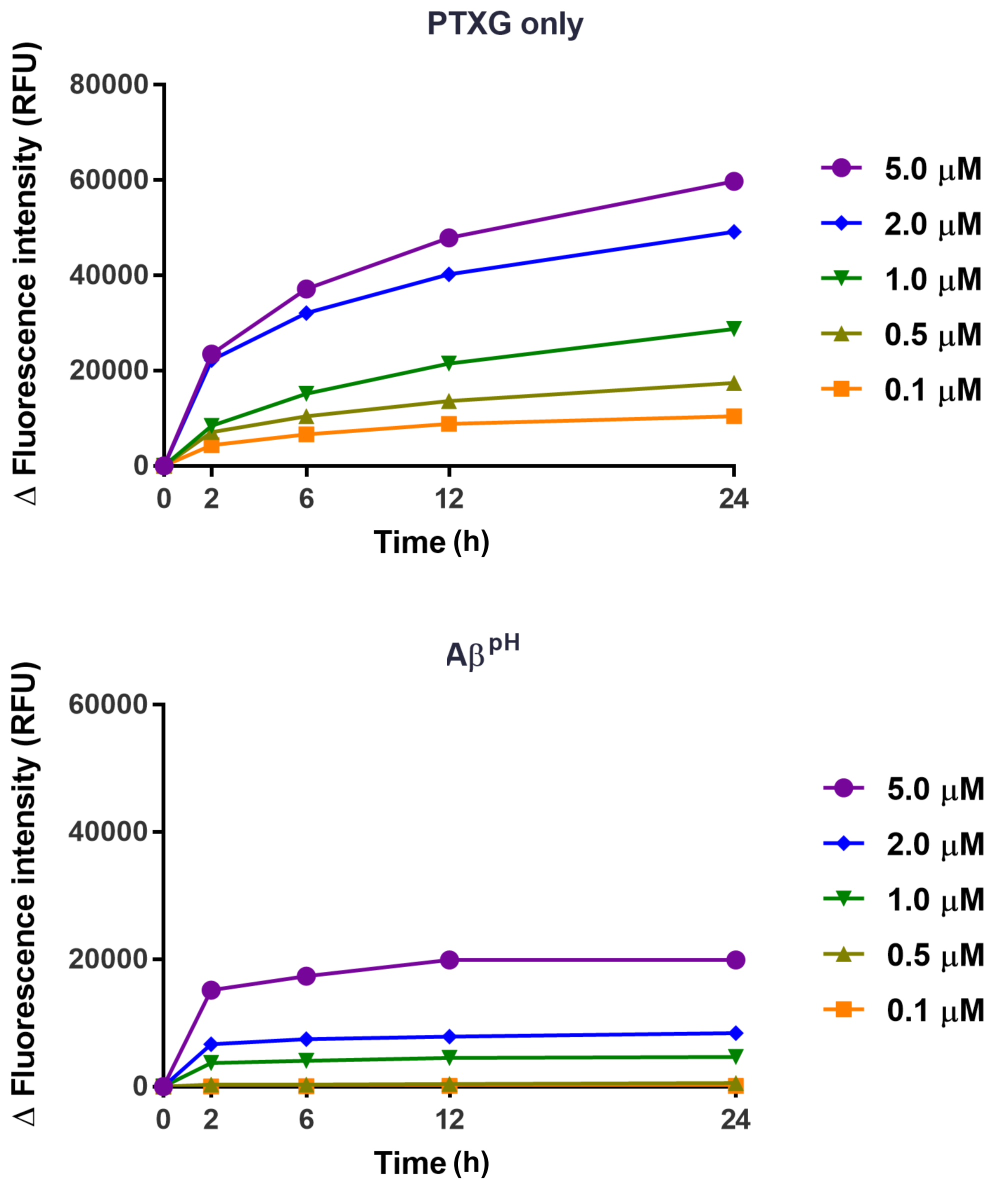

Figure S11. Concentration-dependent response of PTXG and PTXG-conjugated Aβ ($Aβ^{pH}$) at acidic pH over time. Fluorescence intensity of the PTXG-conjugated Aβ ($Aβ^{pH}$, bottom plot) in acidic condition of pH 3.0 over a 24 hour period. Top plot shows the fluorescence intensity of PTXG alone.

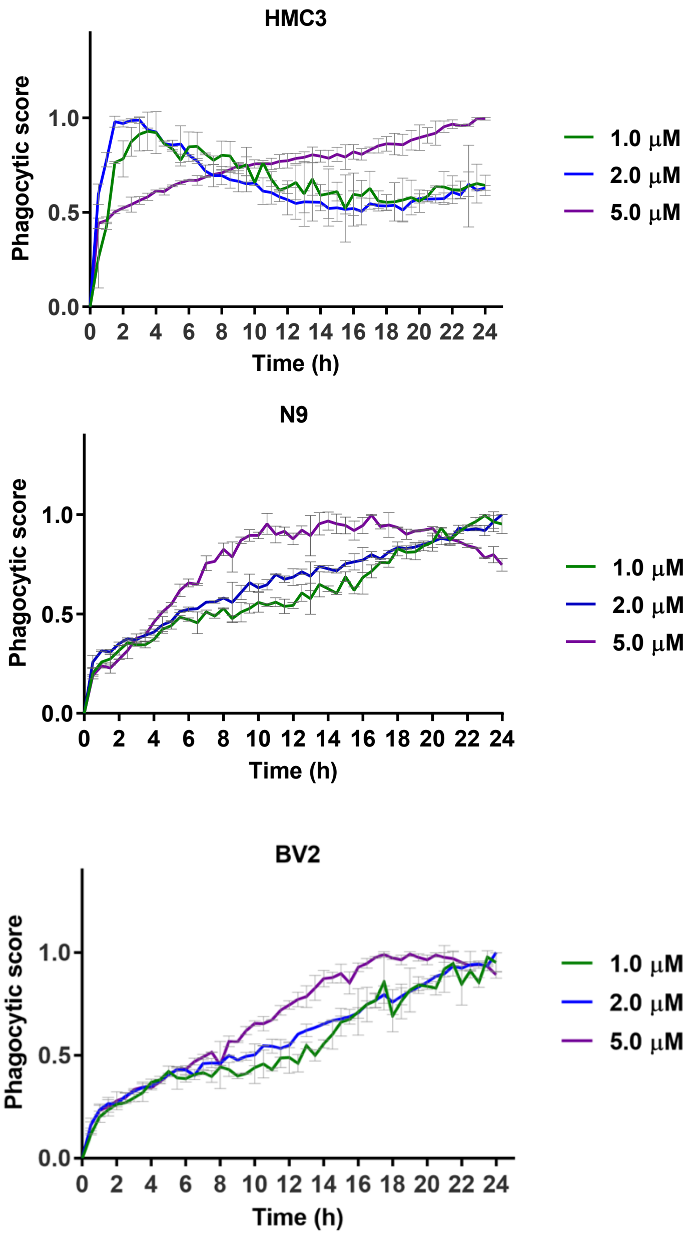

Figure S12. Change in Phagocytic Score over time. For human microglia (HMC3, top), 5 μM of $A\beta^{pH}$ leads to a gradual increase in phagocytic score over time. At lower concentrations of 1 and 2 μM $A\beta^{pH}$, increased phagocytic score is observed peaking between 2 to 6 hours indicating maximum florescence intensity over 24 hours compared to starting (t=0) time point. This is followed by progressive decrease in phagocytic score indicating decreased uptake or increased degradation compared to t=0 time point and ultimately flattening out after 14 hours. In mouse N9 (center) and BV2 (bottom) microglia, higher $A\beta^{pH}$ concentration (5 μM) results in higher phagocytic score over time peaking at 12-16 hours for N9 and 16-20 hours for BV2 compared to no peaks at 1 and 2 μM $A\beta^{pH}$ treatment.

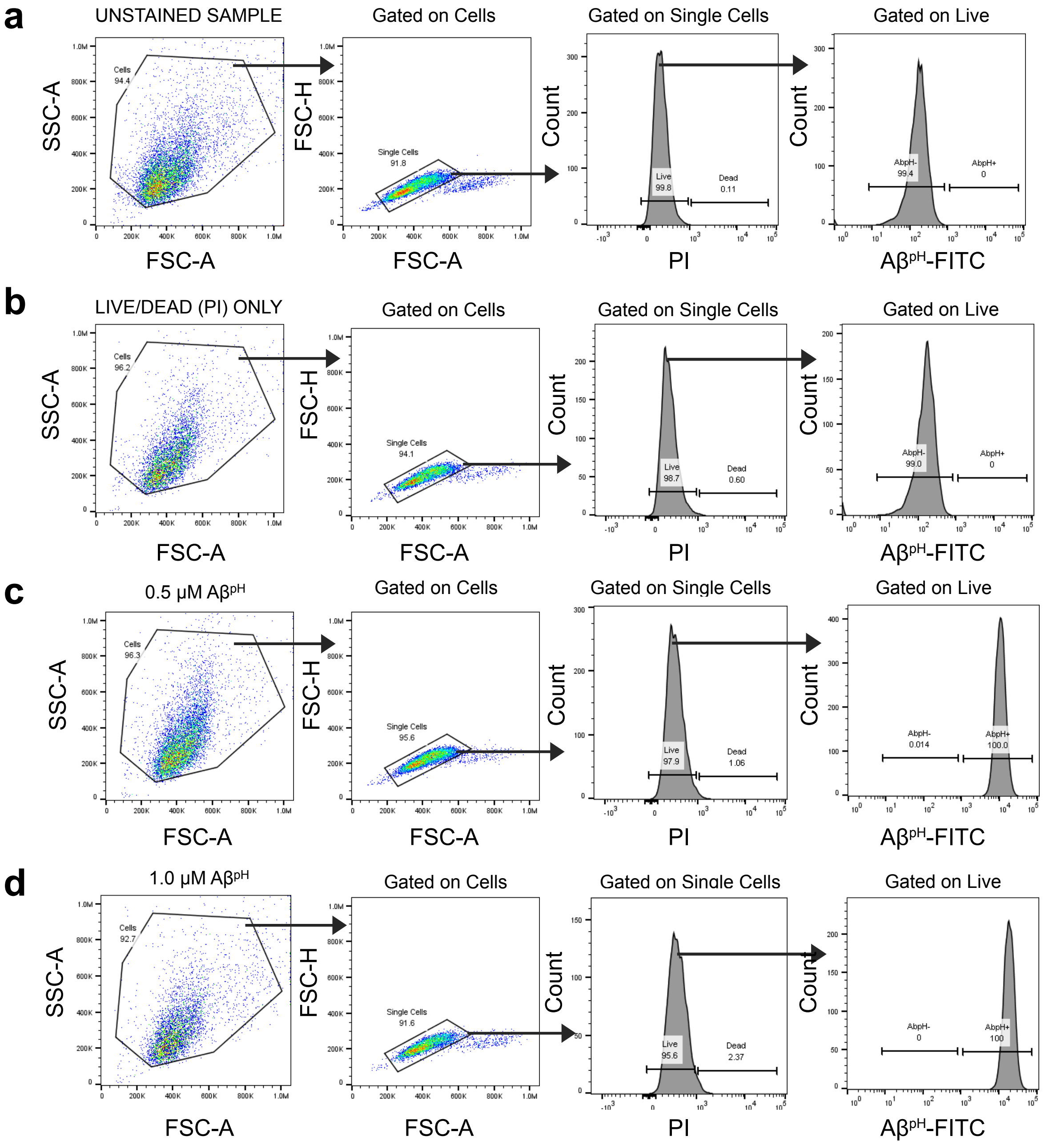

Figure S13A. Gating strategy for flow cytometry analysis of $A\beta^{pH}$ by BV2 microglia. **a.** Unstained sample i.e. cells only. **b.** Cells with live/dead stain only. Propidium iodide (PI) was used as a live/dead stain (indicating dead cells). **c.** Cells treated with 0.5 μM $A\beta^{pH}$ for 1 hour. **d.** Cells treated with 1.0 μM $A\beta^{pH}$ for 1 hour.

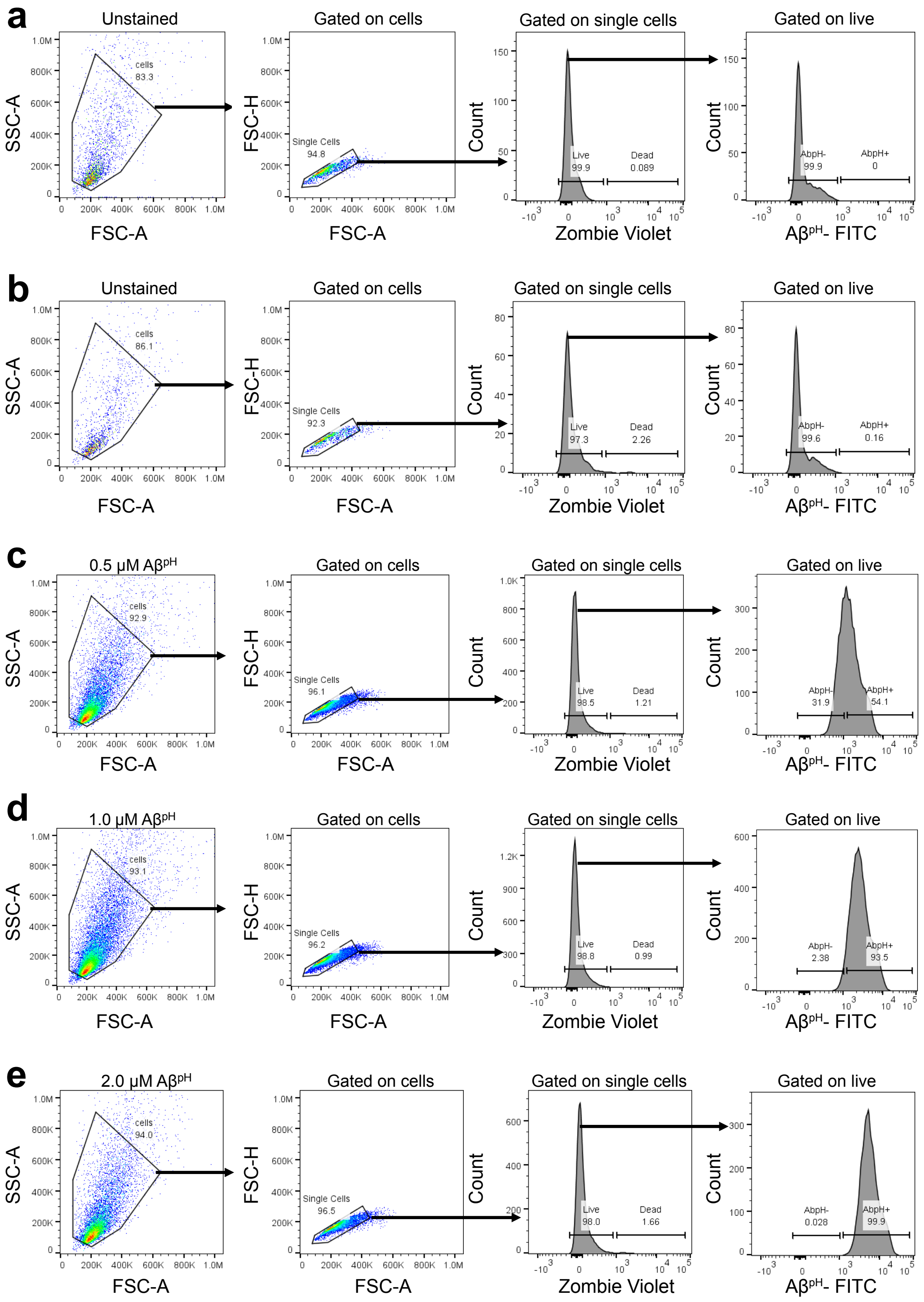

Figure S13B. Gating strategy for flow cytometry analysis of $A\beta^{pH}$ by primary microglia. **a.** Unstained sample i.e. cells only. **b.** Cells with live/dead stain only. Zombie violet was used as a live/dead stain (indicating dead cells). Cells were treated with **c.** 0.5 µM, **d.** 1.0 µM, and **e.** 2.0 µM $A\beta^{pH}$ for 1 hour.

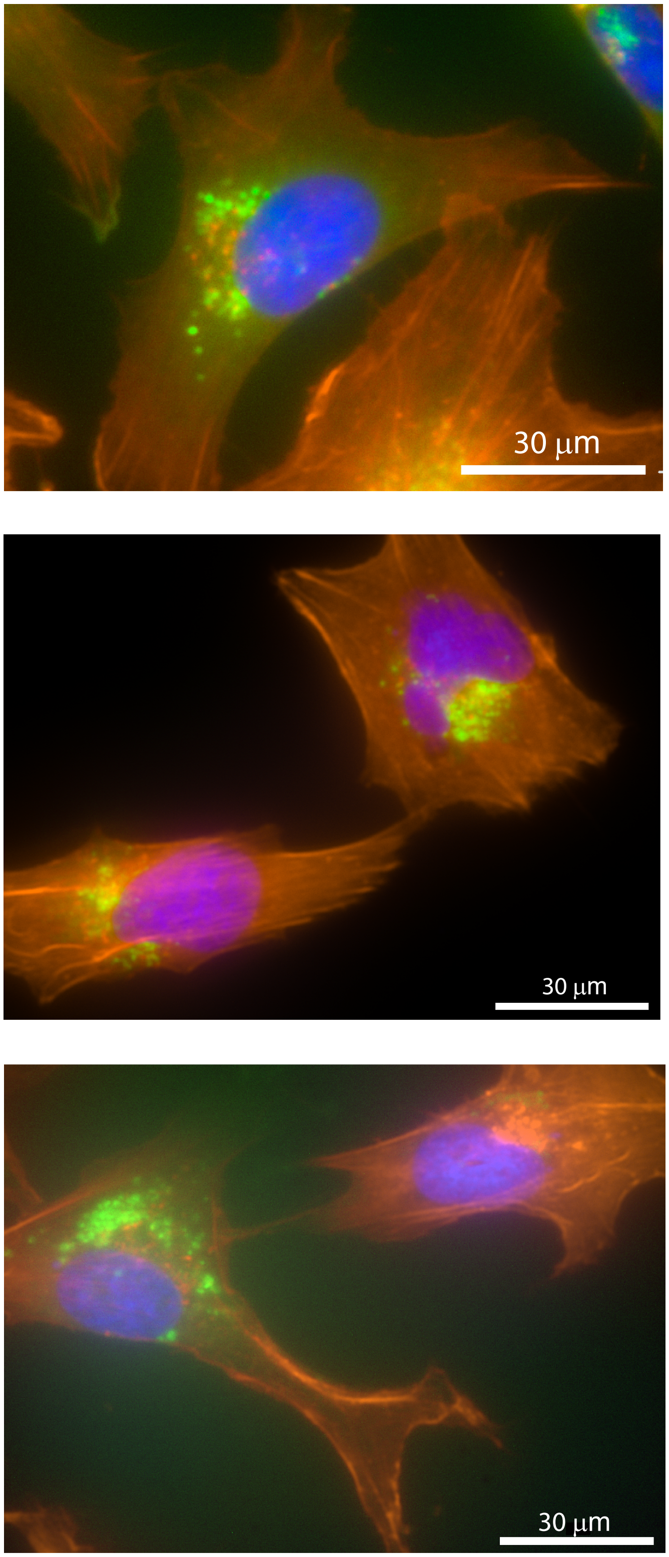

Figure S14A. Imaging phagocytosis of $A\beta^{pH}$ (green) in fixed HMC3 cells stained with phalloidin (red) and DAPI (blue).

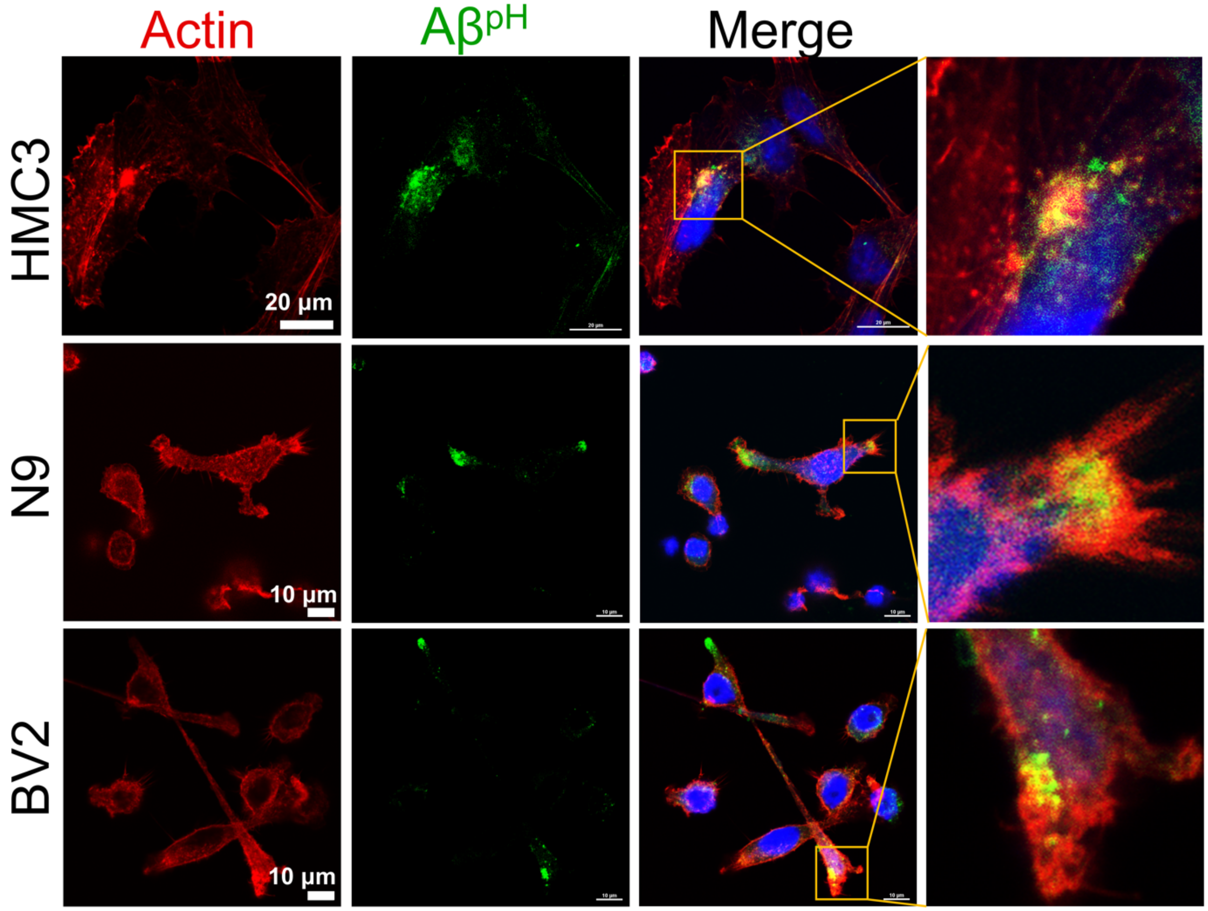

Figure S14B. Confocal images of fixed HMC3, N9, and BV2 cells showing the uptake of $A\beta^{pH}$ (green). Cells are stained for actin (red) and nuclei (blue) and retain the green $A\beta^{pH}$ signal after fixing.

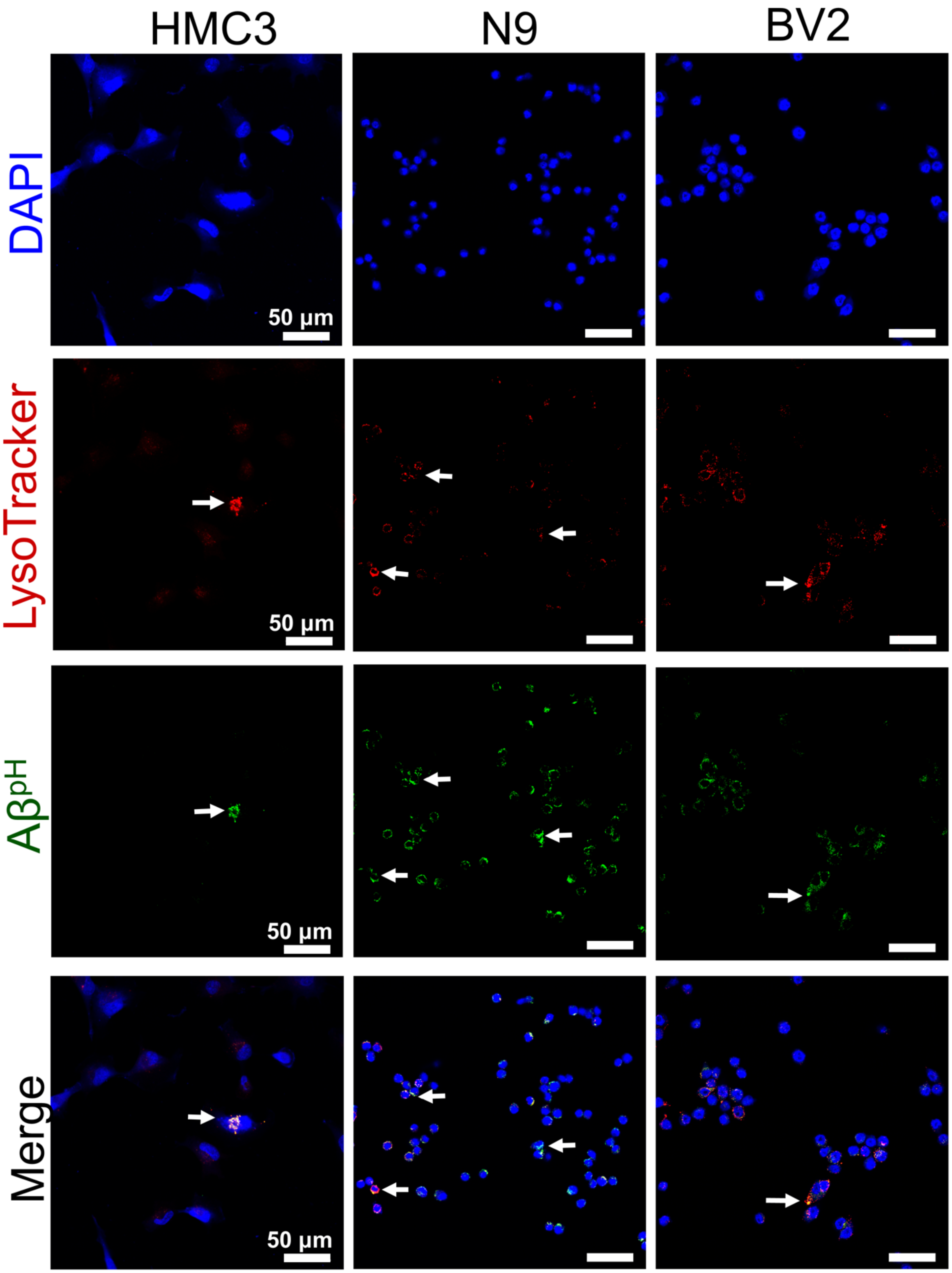

Figure S14C. Confocal images of fixed HMC3, N9, and BV2 cells showing the uptake of $A\beta^{pH}$ (green) as indicated by the white arrows. Cells are stained for acidic intracellular organelles (LysoTracker, red) and nuclei (DAPI, blue).

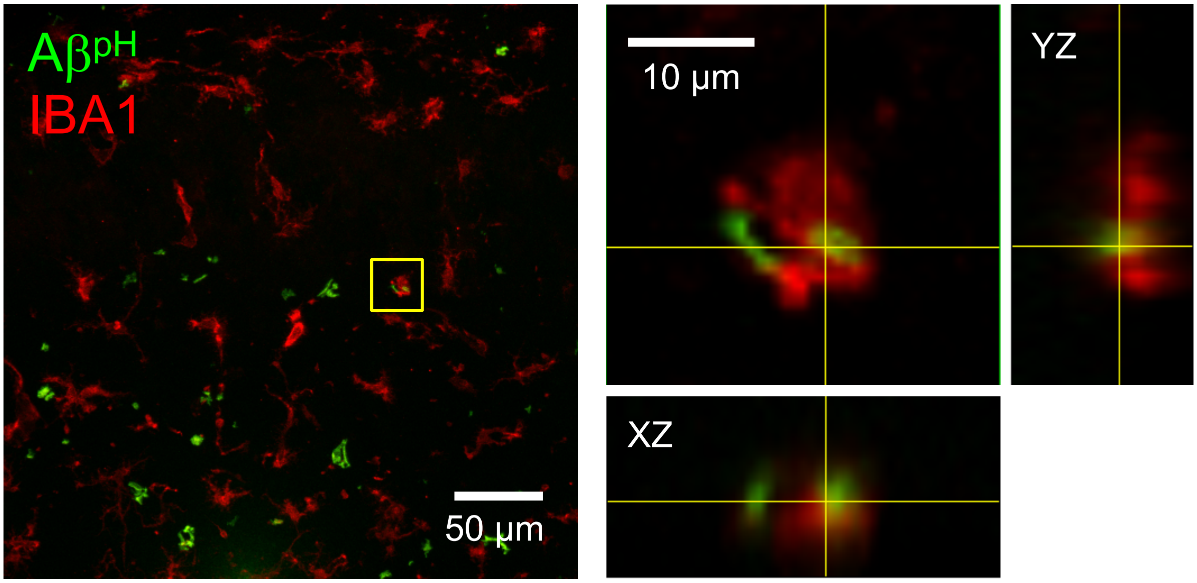

Figure S15. Uptake of $A\beta^{pH}$ (green) by $IBA1^{+}$ microglia (red) in acute hippocampal slices from a P12 rat. The $A\beta^{pH}$ is taken up by microglia shown by overlap of green and red florescence (images on right). Green florescence of $A\beta^{pH}$ outside microglial cells (red) indicates uptake into cells other than microglia.
